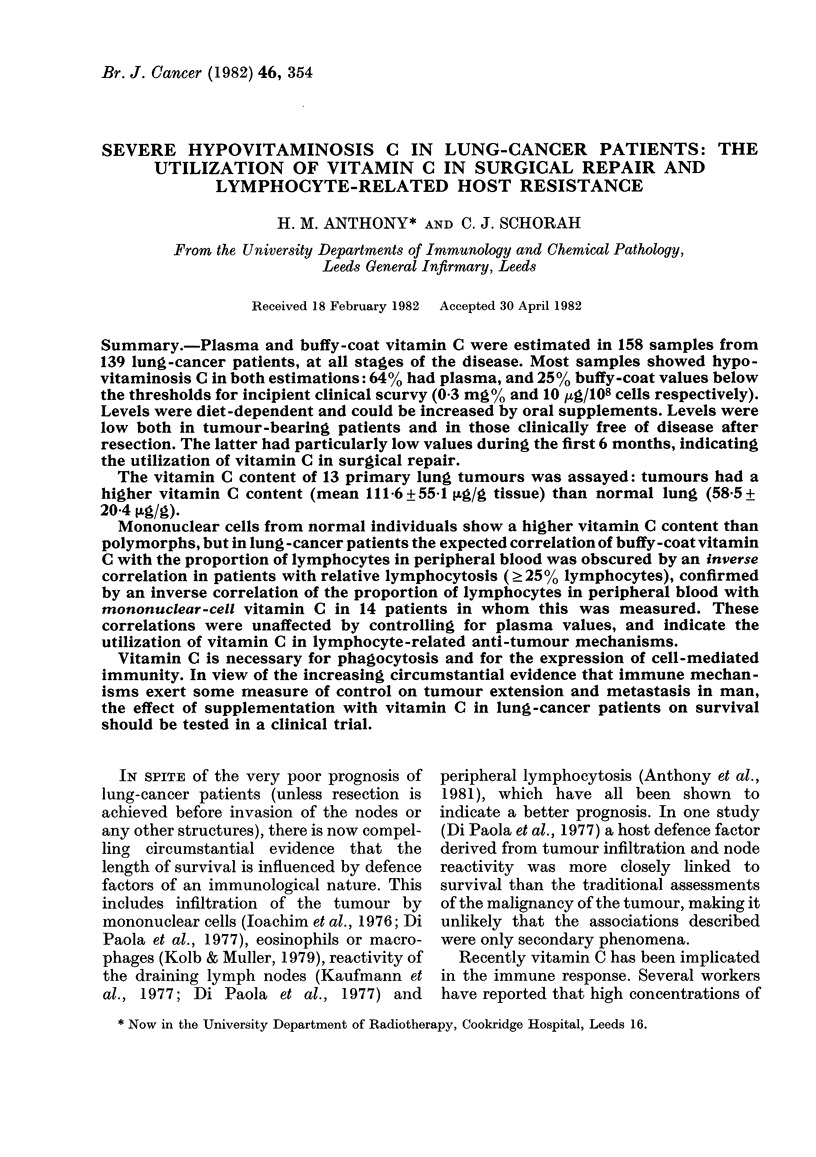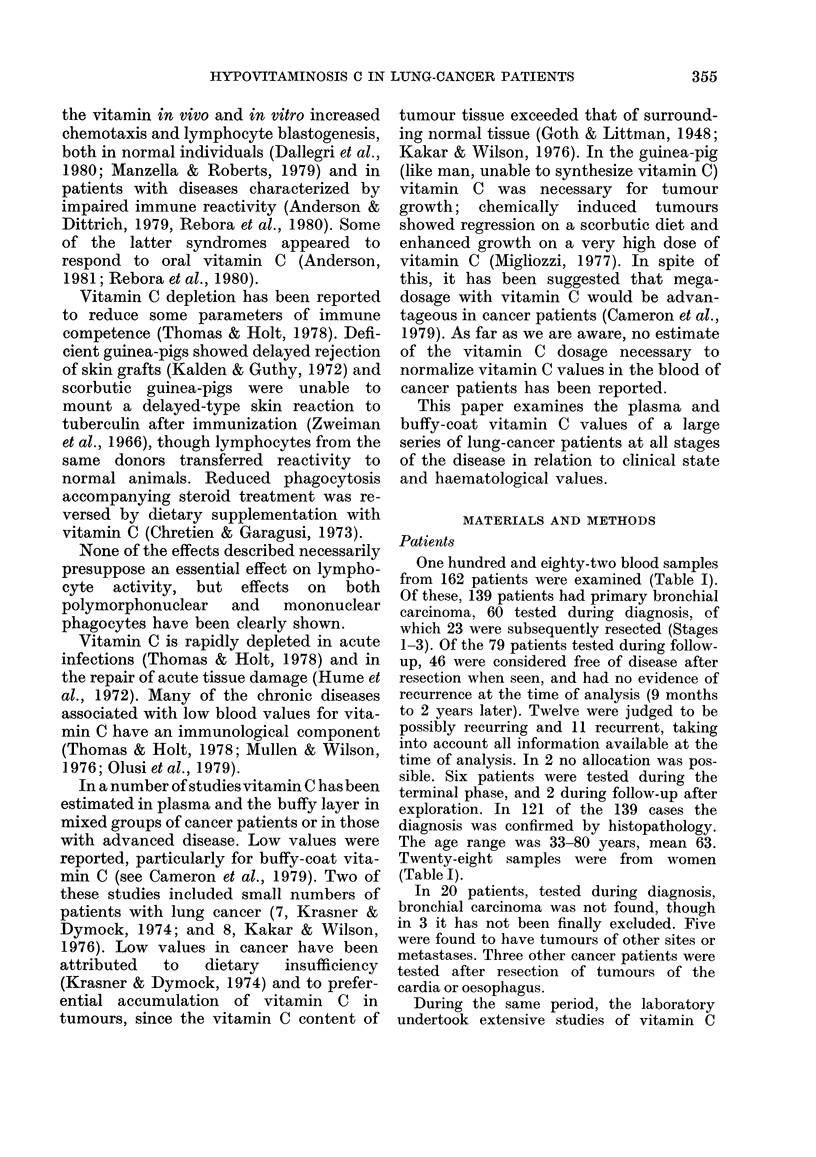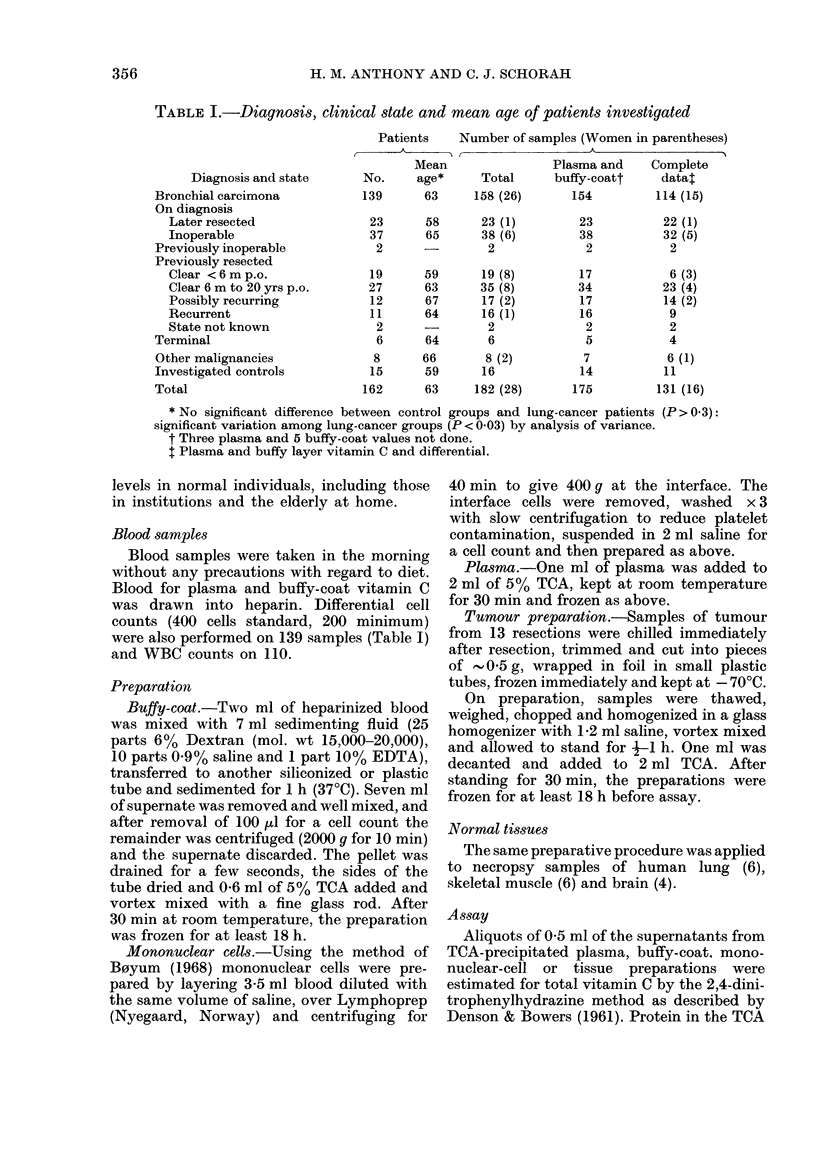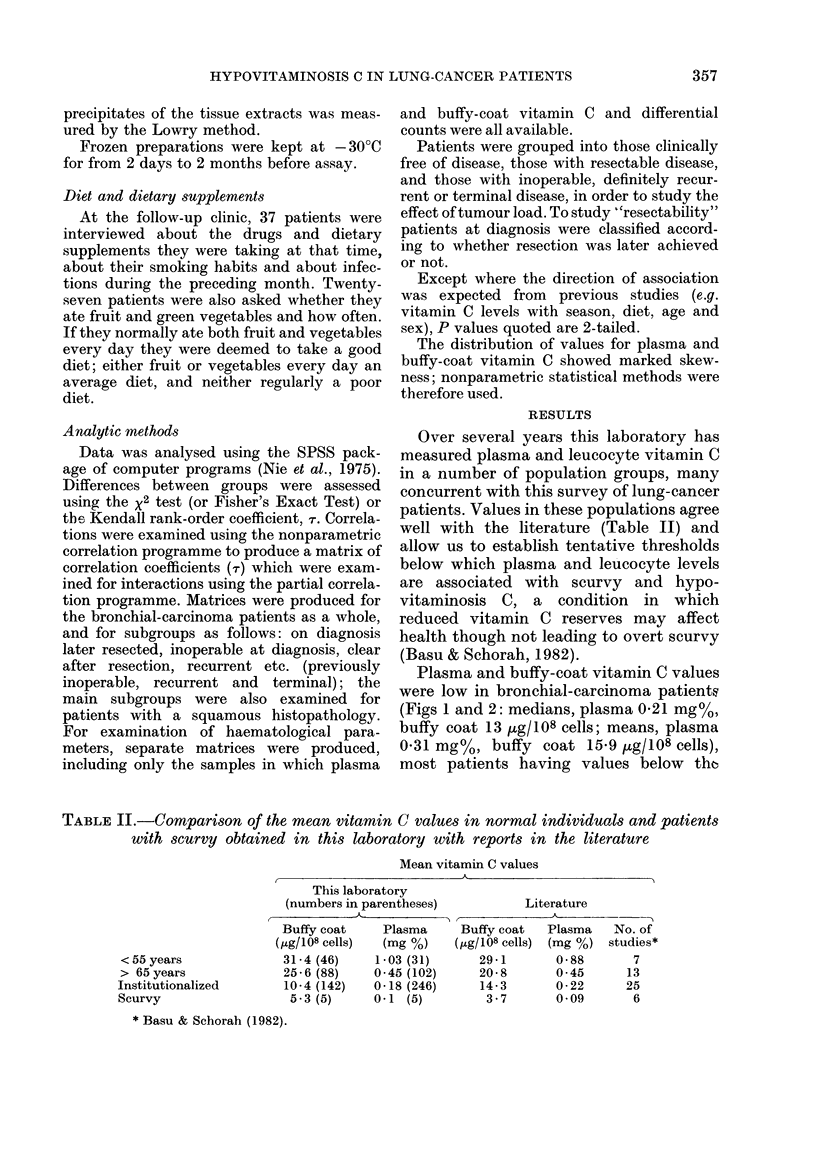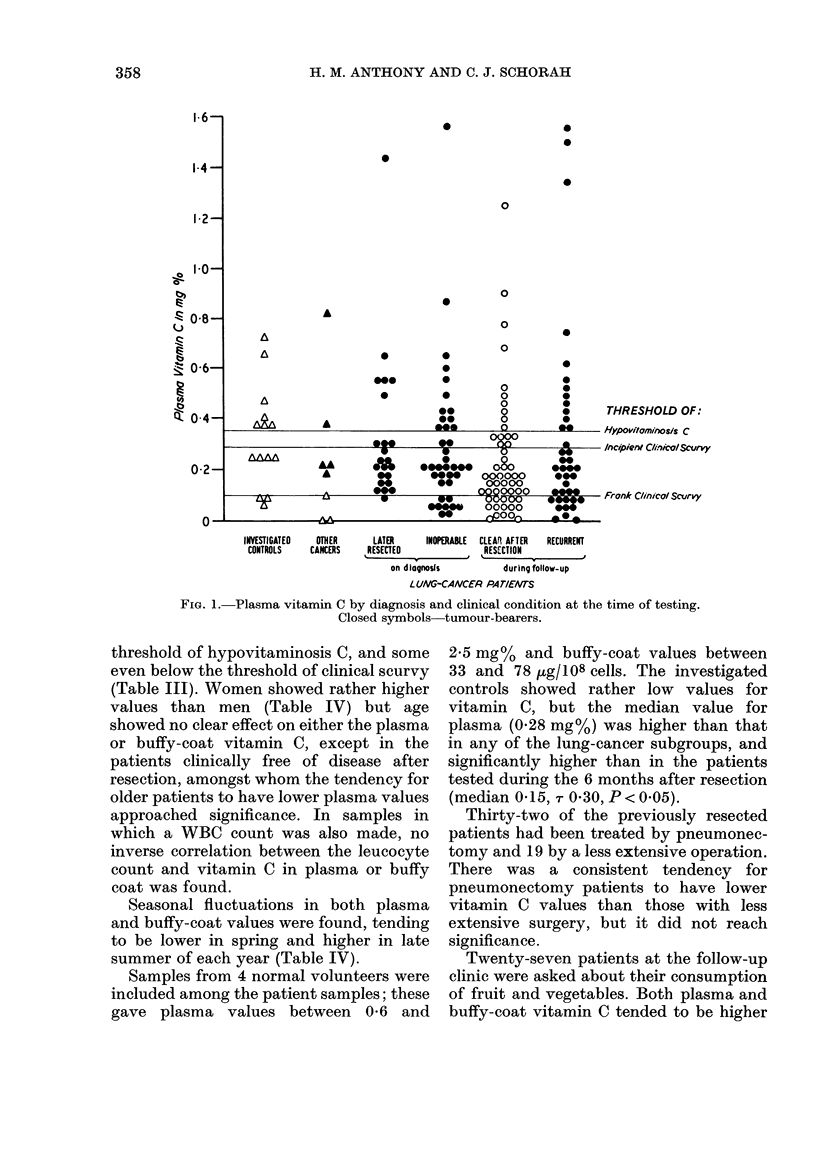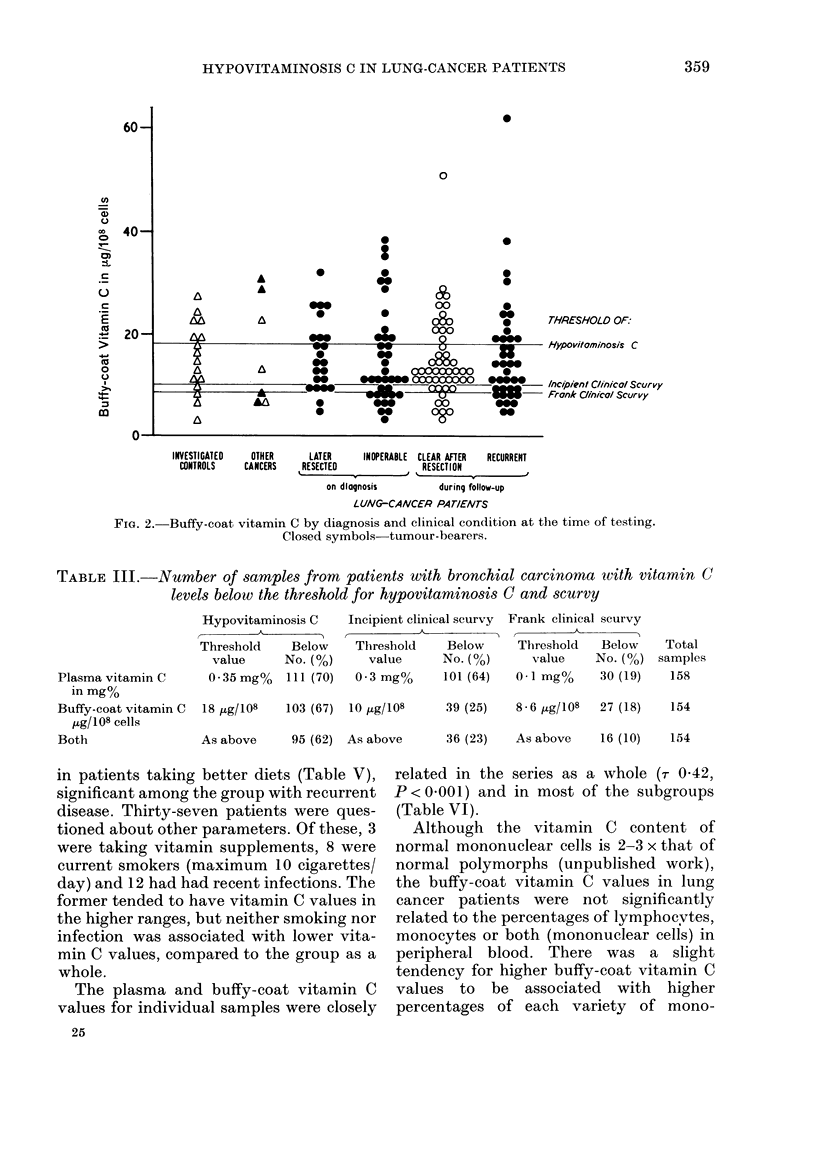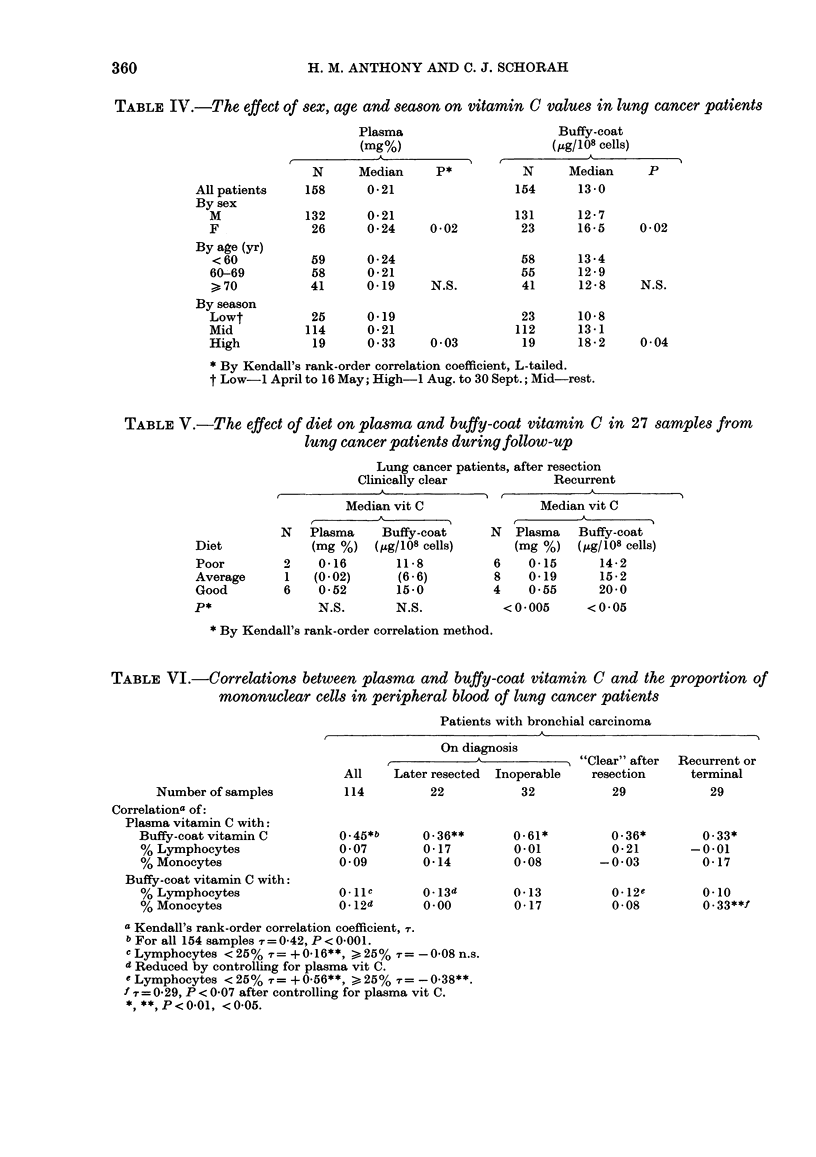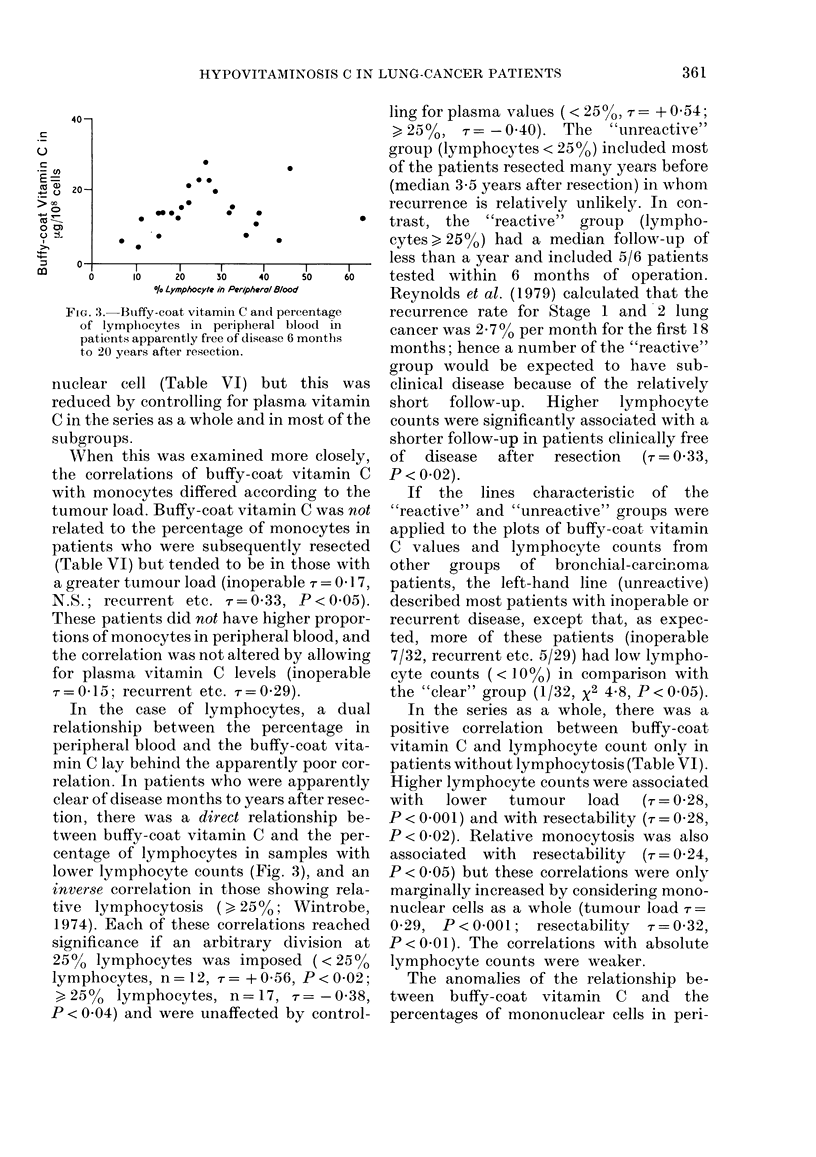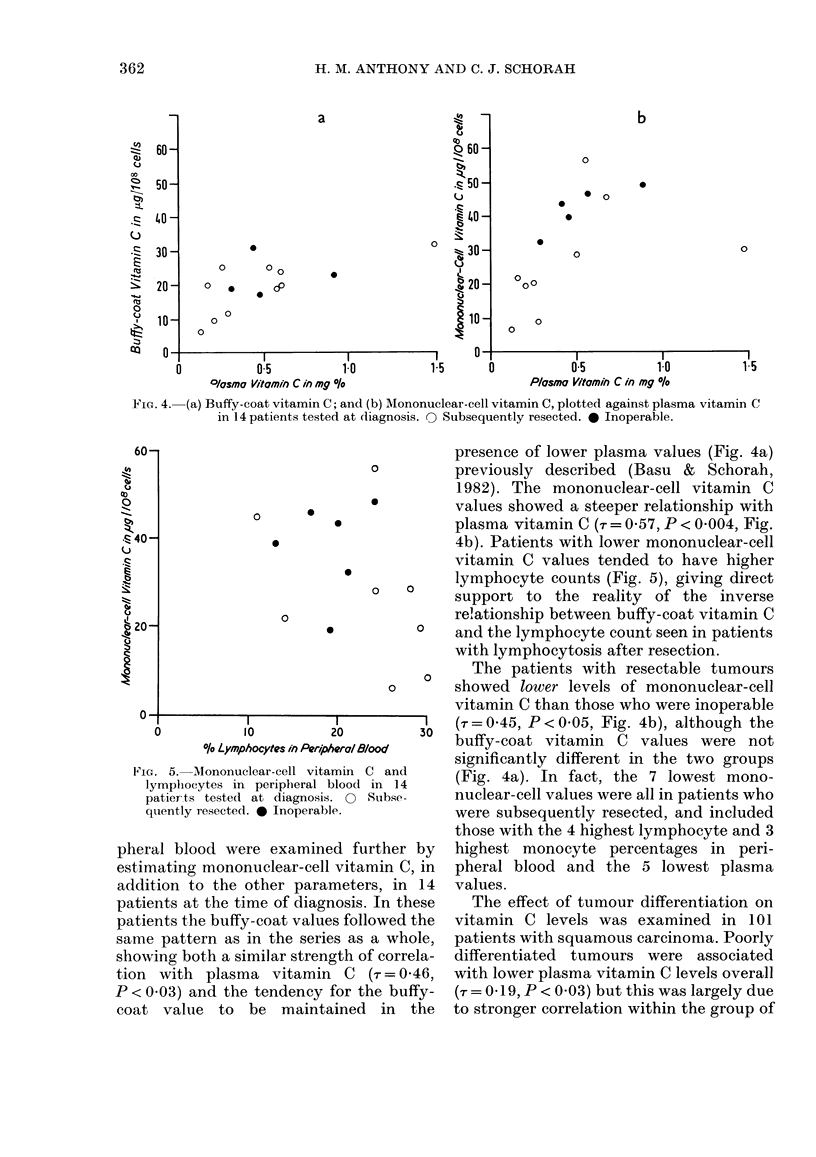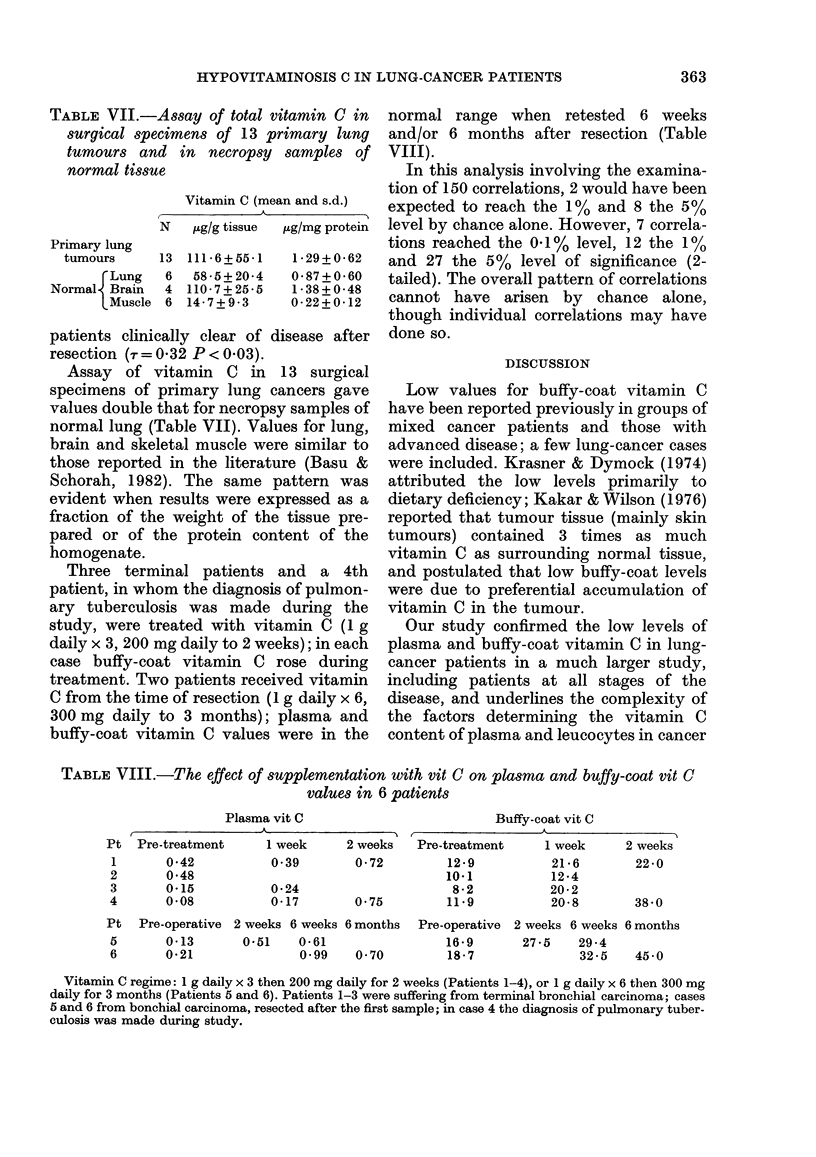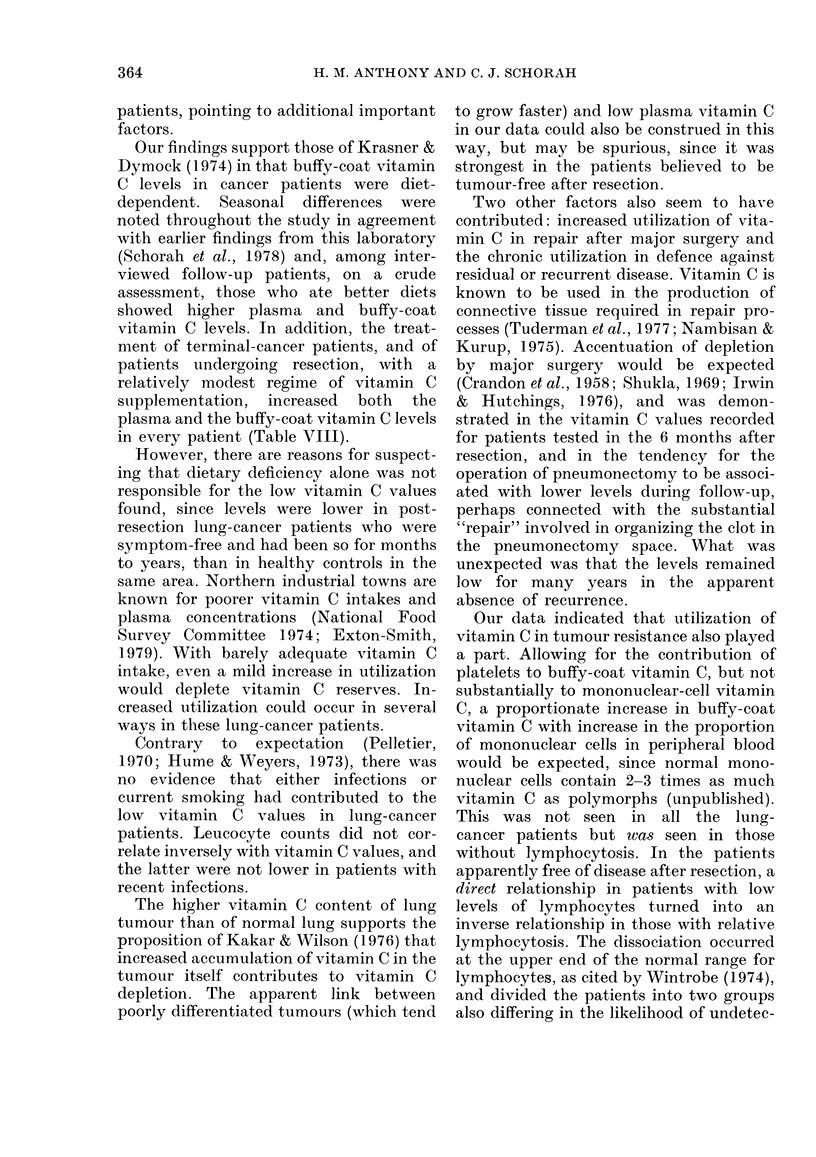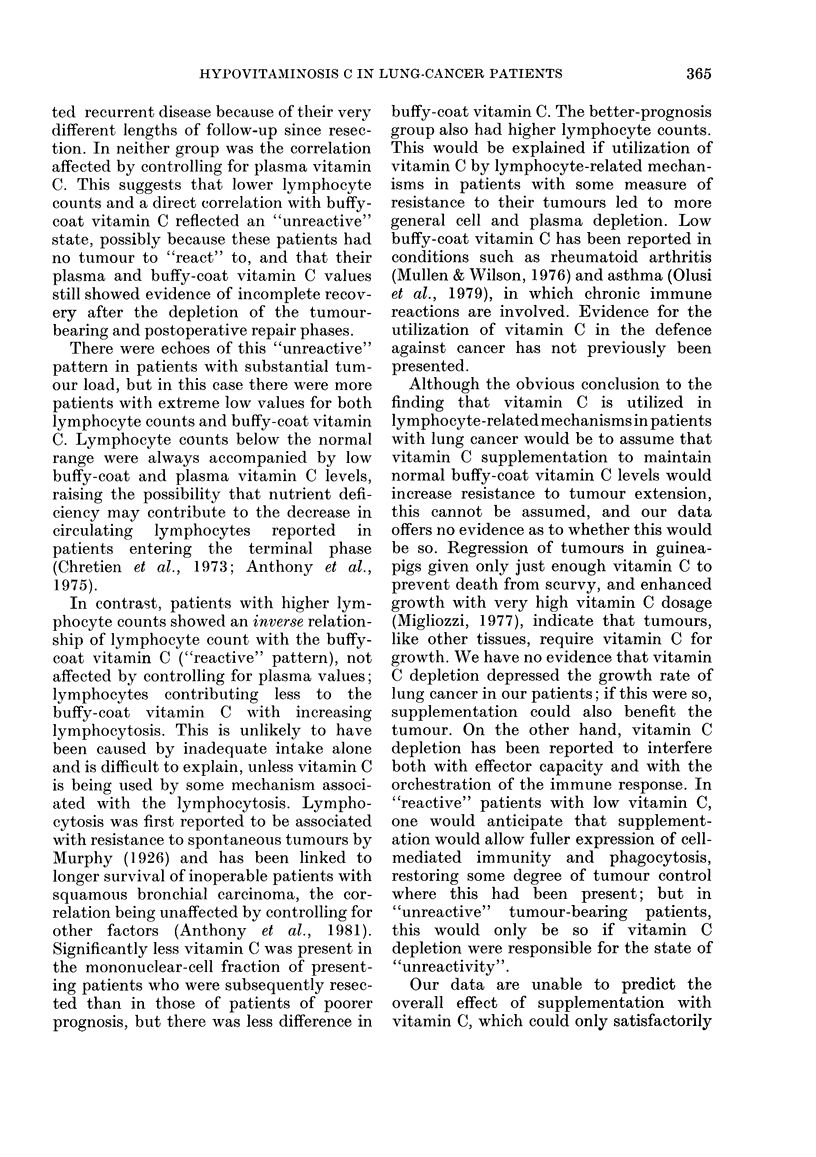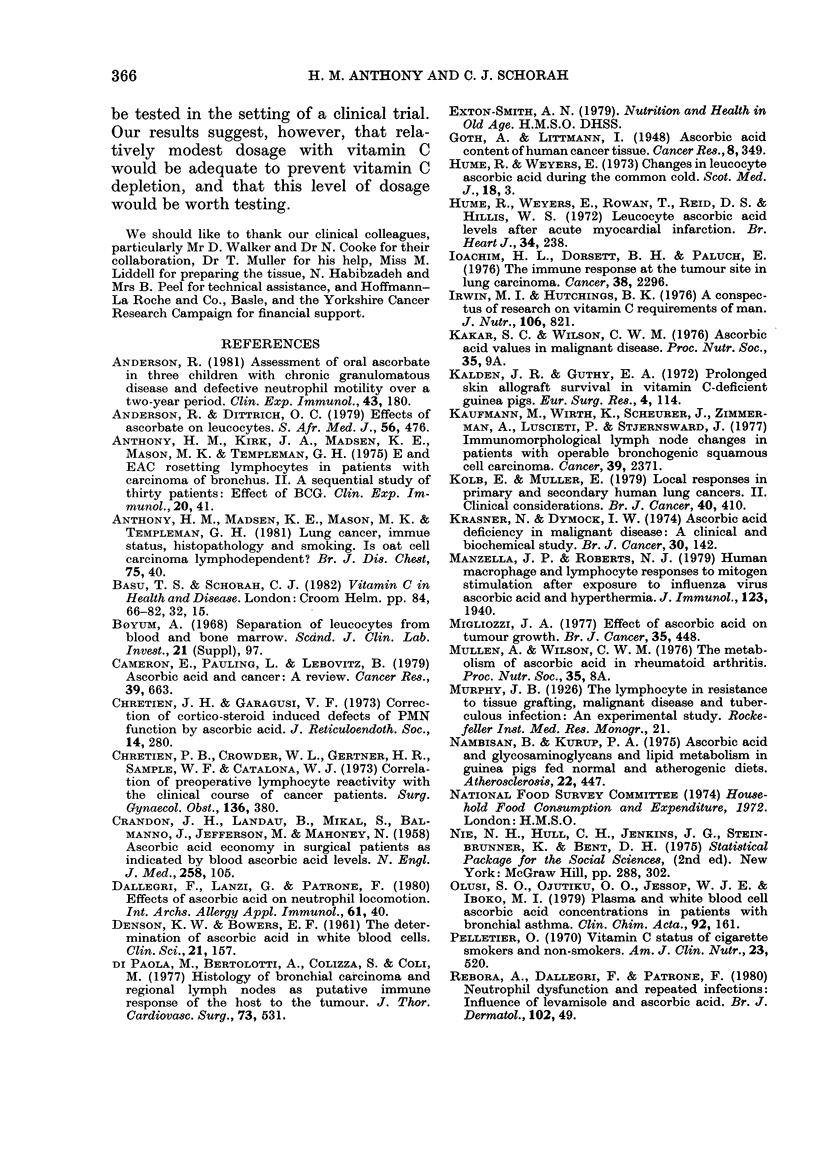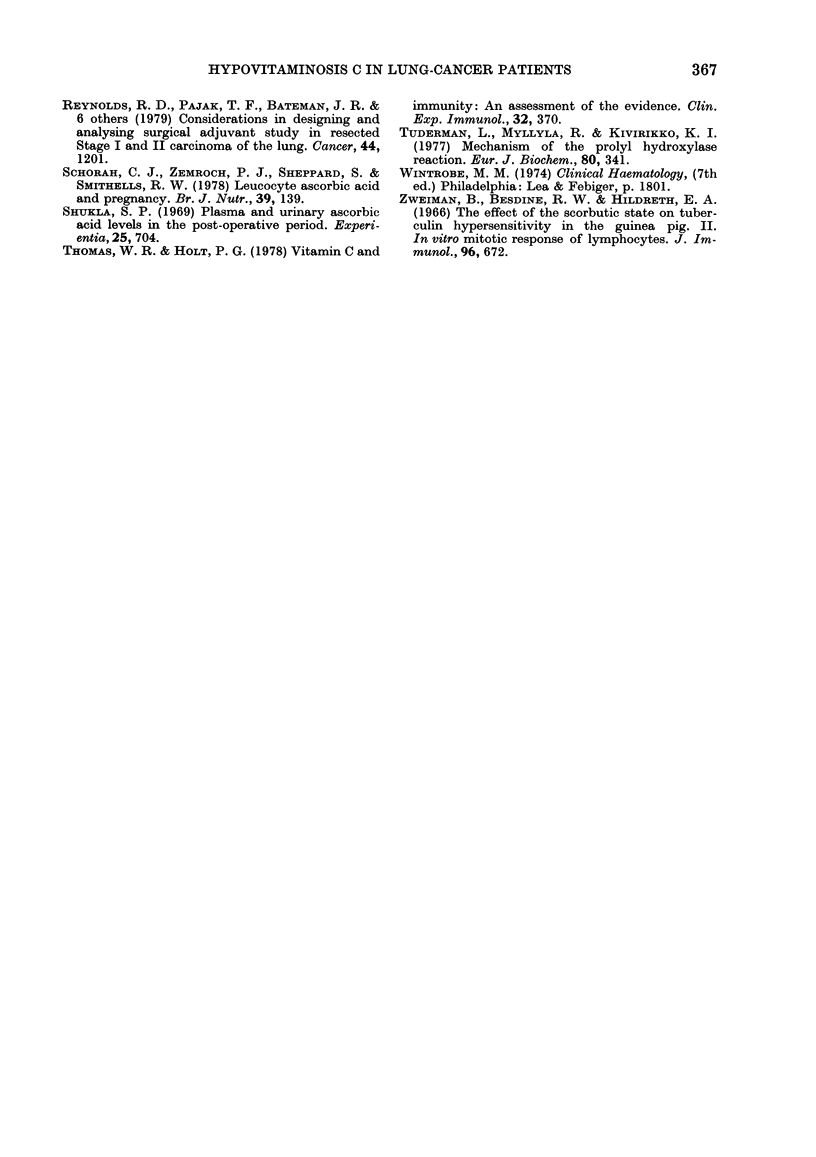# Severe hypovitaminosis C in lung-cancer patients: the utilization of vitamin C in surgical repair and lymphocyte-related host resistance.

**DOI:** 10.1038/bjc.1982.211

**Published:** 1982-09

**Authors:** H. M. Anthony, C. J. Schorah

## Abstract

Plasma and buffy-coat vitamin C were estimated in 158 samples from 139 lung-cancer patients, at all stages of the disease. Most samples showed hypovitaminosis C in both estimations: 64% had plasma, and 25% buffy-coat values below the thresholds for incipient clinical scurvy (0.3 mg% and 10 micrograms/10(8) cells respectively). Levels were diet-dependent and could be increased by oral supplements. Levels were low both in tumour-bearing patients and in those clinically free of disease after resection. The latter had particularly low values during the first 6 months, indicating the utilization of vitamin C in surgical repair. The vitamin C content of 13 primary lung tumours was assayed: tumours had a higher vitamin C content (mean 111.6 +/- 55.1 micrograms/g tissue) than normal lung (58.5 +/- 20.4 micrograms/g). Mononuclear cells from normal individuals show a higher vitamin C content than polymorphs, but in lung-cancer patients the expected correlation of buffy-coat vitamin C with the proportion of lymphocytes in peripheral blood was obscured by an inverse correlation in patients with relative lymphocytosis (greater than or equal to 25% lymphocytes), confirmed by an inverse correlation of the proportion of lymphocytes in peripheral blood with mononuclear-cell vitamin C in 14 patients in whom this was measured. These correlations were unaffected by controlling for plasma values, and indicate the utilization of vitamin C in lymphocyte-related anti-tumour mechanisms. Vitamin C is necessary for phagocytosis and for the expression of cell-mediated immunity. In view of the increasing circumstantial evidence that immune mechanisms exert some measure of control on tumour extension and metastasis in man, the effect of supplementation with vitamin C in lung-cancer patients on survival should be tested in a clinical trial.


*Br. J. Cancer* (1982) **46**, 354

### SEVERE HYPOVITAMINOSIS C IN LUNG-CANCER PATIENTS: THE UTILIZATION OF VITAMIN C IN SURGICAL REPAIR AND LYMPHOCYTE-RELATED HOST RESISTANCE

H. M. ANTHONY* AND C. J. SCHORAH

*From the University Departments of Immunology and Chemical Pathology, Leeds General Infirmary, Leeds*

Received 18 February 1982 Accepted 30 April 1982

**Summary.—Plasma and buffy-coat vitamin C were estimated in 158 samples from 139 lung-cancer patients, at all stages of the disease. Most samples showed hypovitaminosis C in both estimations: 64% had plasma, and 25% buffy-coat values below the thresholds for incipient clinical scurvy (0·3 mg% and 10 μg/$10^8$ cells respectively). Levels were diet-dependent and could be increased by oral supplements. Levels were low both in tumour-bearing patients and in those clinically free of disease after resection. The latter had particularly low values during the first 6 months, indicating the utilization of vitamin C in surgical repair.**

**The vitamin C content of 13 primary lung tumours was assayed: tumours had a higher vitamin C content (mean 111·6 ± 55·1 μg/g tissue) than normal lung (58·5 ± 20·4 μg/g).**

**Mononuclear cells from normal individuals show a higher vitamin C content than polymorphs, but in lung-cancer patients the expected correlation of buffy-coat vitamin C with the proportion of lymphocytes in peripheral blood was obscured by an *inverse* correlation in patients with relative lymphocytosis (≥25% lymphocytes), confirmed by an inverse correlation of the proportion of lymphocytes in peripheral blood with *mononuclear-cell* vitamin C in 14 patients in whom this was measured. These correlations were unaffected by controlling for plasma values, and indicate the utilization of vitamin C in lymphocyte-related anti-tumour mechanisms.**

**Vitamin C is necessary for phagocytosis and for the expression of cell-mediated immunity. In view of the increasing circumstantial evidence that immune mechanisms exert some measure of control on tumour extension and metastasis in man, the effect of supplementation with vitamin C in lung-cancer patients on survival should be tested in a clinical trial.**

IN SPITE of the very poor prognosis of lung-cancer patients (unless resection is achieved before invasion of the nodes or any other structures), there is now compelling circumstantial evidence that the length of survival is influenced by defence factors of an immunological nature. This includes infiltration of the tumour by mononuclear cells (Ioachim *et al.*, 1976; Di Paola *et al.*, 1977), eosinophils or macrophages (Kolb & Muller, 1979), reactivity of the draining lymph nodes (Kaufmann *et al.*, 1977; Di Paola *et al.*, 1977) and peripheral lymphocytosis (Anthony *et al.*, 1981), which have all been shown to indicate a better prognosis. In one study (Di Paola *et al.*, 1977) a host defence factor derived from tumour infiltration and node reactivity was more closely linked to survival than the traditional assessments of the malignancy of the tumour, making it unlikely that the associations described were only secondary phenomena.

Recently vitamin C has been implicated in the immune response. Several workers have reported that high concentrations of

* Now in the University Department of Radiotherapy, Cookridge Hospital, Leeds 16.

the vitamin *in vivo* and *in vitro* increased chemotaxis and lymphocyte blastogenesis, both in normal individuals (Dallegri *et al.*, 1980; Manzella & Roberts, 1979) and in patients with diseases characterized by impaired immune reactivity (Anderson & Dittrich, 1979, Rebora *et al.*, 1980). Some of the latter syndromes appeared to respond to oral vitamin C (Anderson, 1981; Rebora *et al.*, 1980).

Vitamin C depletion has been reported to reduce some parameters of immune competence (Thomas & Holt, 1978). Deficient guinea-pigs showed delayed rejection of skin grafts (Kalden & Guthy, 1972) and scorbutic guinea-pigs were unable to mount a delayed-type skin reaction to tuberculin after immunization (Zweiman *et al.*, 1966), though lymphocytes from the same donors transferred reactivity to normal animals. Reduced phagocytosis accompanying steroid treatment was reversed by dietary supplementation with vitamin C (Chretien & Garagusi, 1973).

None of the effects described necessarily presuppose an essential effect on lymphocyte activity, but effects on both polymorphonuclear and mononuclear phagocytes have been clearly shown.

Vitamin C is rapidly depleted in acute infections (Thomas & Holt, 1978) and in the repair of acute tissue damage (Hume *et al.*, 1972). Many of the chronic diseases associated with low blood values for vitamin C have an immunological component (Thomas & Holt, 1978; Mullen & Wilson, 1976; Olusi *et al.*, 1979).

In a number of studies vitamin C has been estimated in plasma and the buffy layer in mixed groups of cancer patients or in those with advanced disease. Low values were reported, particularly for buffy-coat vitamin C (see Cameron *et al.*, 1979). Two of these studies included small numbers of patients with lung cancer (7, Krasner & Dymock, 1974; and 8, Kakar & Wilson, 1976). Low values in cancer have been attributed to dietary insufficiency (Krasner & Dymock, 1974) and to preferential accumulation of vitamin C in tumours, since the vitamin C content of tumour tissue exceeded that of surrounding normal tissue (Goth & Littman, 1948; Kakar & Wilson, 1976). In the guinea-pig (like man, unable to synthesize vitamin C) vitamin C was necessary for tumour growth; chemically induced tumours showed regression on a scorbutic diet and enhanced growth on a very high dose of vitamin C (Migliozzi, 1977). In spite of this, it has been suggested that megadosage with vitamin C would be advantageous in cancer patients (Cameron *et al.*, 1979). As far as we are aware, no estimate of the vitamin C dosage necessary to normalize vitamin C values in the blood of cancer patients has been reported.

This paper examines the plasma and buffy-coat vitamin C values of a large series of lung-cancer patients at all stages of the disease in relation to clinical state and haematological values.

### MATERIALS AND METHODS

*Patients*

One hundred and eighty-two blood samples from 162 patients were examined (Table I). Of these, 139 patients had primary bronchial carcinoma, 60 tested during diagnosis, of which 23 were subsequently resected (Stages 1–3). Of the 79 patients tested during follow-up, 46 were considered free of disease after resection when seen, and had no evidence of recurrence at the time of analysis (9 months to 2 years later). Twelve were judged to be possibly recurring and 11 recurrent, taking into account all information available at the time of analysis. In 2 no allocation was possible. Six patients were tested during the terminal phase, and 2 during follow-up after exploration. In 121 of the 139 cases the diagnosis was confirmed by histopathology. The age range was 33–80 years, mean 63. Twenty-eight samples were from women (Table I).

In 20 patients, tested during diagnosis, bronchial carcinoma was not found, though in 3 it has not been finally excluded. Five were found to have tumours of other sites or metastases. Three other cancer patients were tested after resection of tumours of the cardia or oesophagus.

During the same period, the laboratory undertook extensive studies of vitamin C

TABLE I.—*Diagnosis, clinical state and mean age of patients investigated*

| Diagnosis and state | Patients: No. | Patients: Mean age* | Number of samples (Women in parentheses): Total | Plasma and buffy-coat† | Complete data‡ |
|---|---|---|---|---|---|
| Bronchial carcimona | 139 | 63 | 158 (26) | 154 | 114 (15) |
| On diagnosis | | | | | |
| Later resected | 23 | 58 | 23 (1) | 23 | 22 (1) |
| Inoperable | 37 | 65 | 38 (6) | 38 | 32 (5) |
| Previously inoperable | 2 | — | 2 | 2 | 2 |
| Previously resected | | | | | |
| Clear < 6 m p.o. | 19 | 59 | 19 (8) | 17 | 6 (3) |
| Clear 6 m to 20 yrs p.o. | 27 | 63 | 35 (8) | 34 | 23 (4) |
| Possibly recurring | 12 | 67 | 17 (2) | 17 | 14 (2) |
| Recurrent | 11 | 64 | 16 (1) | 16 | 9 |
| State not known | 2 | — | 2 | 2 | 2 |
| Terminal | 6 | 64 | 6 | 5 | 4 |
| Other malignancies | 8 | 66 | 8 (2) | 7 | 6 (1) |
| Investigated controls | 15 | 59 | 16 | 14 | 11 |
| Total | 162 | 63 | 182 (28) | 175 | 131 (16) |

* No significant difference between control groups and lung-cancer patients ($P > 0{\cdot}3$): significant variation among lung-cancer groups ($P < 0{\cdot}03$) by analysis of variance.

† Three plasma and 5 buffy-coat values not done.

‡ Plasma and buffy layer vitamin C and differential.

levels in normal individuals, including those in institutions and the elderly at home.

*Blood samples*

Blood samples were taken in the morning without any precautions with regard to diet. Blood for plasma and buffy-coat vitamin C was drawn into heparin. Differential cell counts (400 cells standard, 200 minimum) were also performed on 139 samples (Table I) and WBC counts on 110.

*Preparation*

*Buffy-coat.*—Two ml of heparinized blood was mixed with 7 ml sedimenting fluid (25 parts 6% Dextran (mol. wt 15,000–20,000), 10 parts 0·9% saline and 1 part 10% EDTA), transferred to another siliconized or plastic tube and sedimented for 1 h (37°C). Seven ml of supernate was removed and well mixed, and after removal of 100 $\mu$l for a cell count the remainder was centrifuged (2000 $g$ for 10 min) and the supernate discarded. The pellet was drained for a few seconds, the sides of the tube dried and 0·6 ml of 5% TCA added and vortex mixed with a fine glass rod. After 30 min at room temperature, the preparation was frozen for at least 18 h.

*Mononuclear cells.*—Using the method of Bøyum (1968) mononuclear cells were prepared by layering 3·5 ml blood diluted with the same volume of saline, over Lymphoprep (Nyegaard, Norway) and centrifuging for 40 min to give 400 $g$ at the interface. The interface cells were removed, washed ×3 with slow centrifugation to reduce platelet contamination, suspended in 2 ml saline for a cell count and then prepared as above.

*Plasma.*—One ml of plasma was added to 2 ml of 5% TCA, kept at room temperature for 30 min and frozen as above.

*Tumour preparation.*—Samples of tumour from 13 resections were chilled immediately after resection, trimmed and cut into pieces of ~0·5 g, wrapped in foil in small plastic tubes, frozen immediately and kept at −70°C.

On preparation, samples were thawed, weighed, chopped and homogenized in a glass homogenizer with 1·2 ml saline, vortex mixed and allowed to stand for ½–1 h. One ml was decanted and added to 2 ml TCA. After standing for 30 min, the preparations were frozen for at least 18 h before assay.

*Normal tissues*

The same preparative procedure was applied to necropsy samples of human lung (6), skeletal muscle (6) and brain (4).

*Assay*

Aliquots of 0·5 ml of the supernatants from TCA-precipitated plasma, buffy-coat, mononuclear-cell or tissue preparations were estimated for total vitamin C by the 2,4-dinitrophenylhydrazine method as described by Denson & Bowers (1961). Protein in the TCA

precipitates of the tissue extracts was measured by the Lowry method.

Frozen preparations were kept at −30°C for from 2 days to 2 months before assay.

*Diet and dietary supplements*

At the follow-up clinic, 37 patients were interviewed about the drugs and dietary supplements they were taking at that time, about their smoking habits and about infections during the preceding month. Twenty-seven patients were also asked whether they ate fruit and green vegetables and how often. If they normally ate both fruit and vegetables every day they were deemed to take a good diet; either fruit or vegetables every day an average diet, and neither regularly a poor diet.

*Analytic methods*

Data was analysed using the SPSS package of computer programs (Nie *et al.*, 1975). Differences between groups were assessed using the $\chi^2$ test (or Fisher's Exact Test) or the Kendall rank-order coefficient, $\tau$. Correlations were examined using the nonparametric correlation programme to produce a matrix of correlation coefficients ($\tau$) which were examined for interactions using the partial correlation programme. Matrices were produced for the bronchial-carcinoma patients as a whole, and for subgroups as follows: on diagnosis later resected, inoperable at diagnosis, clear after resection, recurrent etc. (previously inoperable, recurrent and terminal); the main subgroups were also examined for patients with a squamous histopathology. For examination of haematological parameters, separate matrices were produced, including only the samples in which plasma and buffy-coat vitamin C and differential counts were all available.

Patients were grouped into those clinically free of disease, those with resectable disease, and those with inoperable, definitely recurrent or terminal disease, in order to study the effect of tumour load. To study "resectability" patients at diagnosis were classified according to whether resection was later achieved or not.

Except where the direction of association was expected from previous studies (*e.g.* vitamin C levels with season, diet, age and sex), $P$ values quoted are 2-tailed.

The distribution of values for plasma and buffy-coat vitamin C showed marked skewness; nonparametric statistical methods were therefore used.

### RESULTS

Over several years this laboratory has measured plasma and leucocyte vitamin C in a number of population groups, many concurrent with this survey of lung-cancer patients. Values in these populations agree well with the literature (Table II) and allow us to establish tentative thresholds below which plasma and leucocyte levels are associated with scurvy and hypovitaminosis C, a condition in which reduced vitamin C reserves may affect health though not leading to overt scurvy (Basu & Schorah, 1982).

Plasma and buffy-coat vitamin C values were low in bronchial-carcinoma patients (Figs 1 and 2: medians, plasma 0·21 mg%, buffy coat 13 $\mu$g/$10^8$ cells; means, plasma 0·31 mg%, buffy coat 15·9 $\mu$g/$10^8$ cells), most patients having values below the

TABLE II.—*Comparison of the mean vitamin C values in normal individuals and patients with scurvy obtained in this laboratory with reports in the literature*

| | Mean vitamin C values | | | | |
|---|---|---|---|---|---|
| | This laboratory (numbers in parentheses) | | Literature | | |
| | Buffy coat ($\mu$g/$10^8$ cells) | Plasma (mg %) | Buffy coat ($\mu$g/$10^8$ cells) | Plasma (mg %) | No. of studies* |
| < 55 years | 31·4 (46) | 1·03 (31) | 29·1 | 0·88 | 7 |
| > 65 years | 25·6 (88) | 0·45 (102) | 20·8 | 0·45 | 13 |
| Institutionalized | 10·4 (142) | 0·18 (246) | 14·3 | 0·22 | 25 |
| Scurvy | 5·3 (5) | 0·1 (5) | 3·7 | 0·09 | 6 |

* Basu & Schorah (1982).

FIG. 1.—Plasma vitamin C by diagnosis and clinical condition at the time of testing. Closed symbols—tumour-bearers.

threshold of hypovitaminosis C, and some even below the threshold of clinical scurvy (Table III). Women showed rather higher values than men (Table IV) but age showed no clear effect on either the plasma or buffy-coat vitamin C, except in the patients clinically free of disease after resection, amongst whom the tendency for older patients to have lower plasma values approached significance. In samples in which a WBC count was also made, no inverse correlation between the leucocyte count and vitamin C in plasma or buffy coat was found.

Seasonal fluctuations in both plasma and buffy-coat values were found, tending to be lower in spring and higher in late summer of each year (Table IV).

Samples from 4 normal volunteers were included among the patient samples; these gave plasma values between 0·6 and 2·5 mg% and buffy-coat values between 33 and 78 $\mu g/10^8$ cells. The investigated controls showed rather low values for vitamin C, but the median value for plasma (0·28 mg%) was higher than that in any of the lung-cancer subgroups, and significantly higher than in the patients tested during the 6 months after resection (median 0·15, $\tau$ 0·30, $P < 0{\cdot}05$).

Thirty-two of the previously resected patients had been treated by pneumonectomy and 19 by a less extensive operation. There was a consistent tendency for pneumonectomy patients to have lower vitamin C values than those with less extensive surgery, but it did not reach significance.

Twenty-seven patients at the follow-up clinic were asked about their consumption of fruit and vegetables. Both plasma and buffy-coat vitamin C tended to be higher

FIG. 2.—Buffy-coat vitamin C by diagnosis and clinical condition at the time of testing. Closed symbols—tumour-bearers.

TABLE III.—*Number of samples from patients with bronchial carcinoma with vitamin C levels below the threshold for hypovitaminosis C and scurvy*

| | Hypovitaminosis C | | Incipient clinical scurvy | | Frank clinical scurvy | | |
|---|---|---|---|---|---|---|---|
| | Threshold value | Below No. (%) | Threshold value | Below No. (%) | Threshold value | Below No. (%) | Total samples |
| Plasma vitamin C in mg% | 0·35 mg% | 111 (70) | 0·3 mg% | 101 (64) | 0·1 mg% | 30 (19) | 158 |
| Buffy-coat vitamin C μg/10⁸ cells | 18 μg/10⁸ | 103 (67) | 10 μg/10⁸ | 39 (25) | 8·6 μg/10⁸ | 27 (18) | 154 |
| Both | As above | 95 (62) | As above | 36 (23) | As above | 16 (10) | 154 |

in patients taking better diets (Table V), significant among the group with recurrent disease. Thirty-seven patients were questioned about other parameters. Of these, 3 were taking vitamin supplements, 8 were current smokers (maximum 10 cigarettes/day) and 12 had had recent infections. The former tended to have vitamin C values in the higher ranges, but neither smoking nor infection was associated with lower vitamin C values, compared to the group as a whole.

The plasma and buffy-coat vitamin C values for individual samples were closely related in the series as a whole ($\tau$ 0·42, $P < 0{\cdot}001$) and in most of the subgroups (Table VI).

Although the vitamin C content of normal mononuclear cells is 2–3 × that of normal polymorphs (unpublished work), the buffy-coat vitamin C values in lung cancer patients were not significantly related to the percentages of lymphocytes, monocytes or both (mononuclear cells) in peripheral blood. There was a slight tendency for higher buffy-coat vitamin C values to be associated with higher percentages of each variety of mono-

TABLE IV.—*The effect of sex, age and season on vitamin C values in lung cancer patients*

| | Plasma (mg%) | | | Buffy-coat (μg/10[8] cells) | | |
|---|---|---|---|---|---|---|
| | N | Median | P* | N | Median | P |
| All patients | 158 | 0·21 | | 154 | 13·0 | |
| By sex | | | | | | |
| M | 132 | 0·21 | | 131 | 12·7 | |
| F | 26 | 0·24 | 0·02 | 23 | 16·5 | 0·02 |
| By age (yr) | | | | | | |
| < 60 | 59 | 0·24 | | 58 | 13·4 | |
| 60–69 | 58 | 0·21 | | 55 | 12·9 | |
| ⩾70 | 41 | 0·19 | N.S. | 41 | 12·8 | N.S. |
| By season | | | | | | |
| Low† | 25 | 0·19 | | 23 | 10·8 | |
| Mid | 114 | 0·21 | | 112 | 13·1 | |
| High | 19 | 0·33 | 0·03 | 19 | 18·2 | 0·04 |

* By Kendall's rank-order correlation coefficient, L-tailed.
† Low—1 April to 16 May; High—1 Aug. to 30 Sept.; Mid—rest.

TABLE V.—*The effect of diet on plasma and buffy-coat vitamin C in 27 samples from lung cancer patients during follow-up*

| | Lung cancer patients, after resection: Clinically clear | | | Recurrent | | |
|---|---|---|---|---|---|---|
| | | Median vit C | | | Median vit C | |
| Diet | N | Plasma (mg %) | Buffy-coat (μg/10[8] cells) | N | Plasma (mg %) | Buffy-coat (μg/10[8] cells) |
| Poor | 2 | 0·16 | 11·8 | 6 | 0·15 | 14·2 |
| Average | 1 | (0·02) | (6·6) | 8 | 0·19 | 15·2 |
| Good | 6 | 0·52 | 15·0 | 4 | 0·55 | 20·0 |
| *P** | | N.S. | N.S. | | < 0·005 | < 0·05 |

* By Kendall's rank-order correlation method.

TABLE VI.—*Correlations between plasma and buffy-coat vitamin C and the proportion of mononuclear cells in peripheral blood of lung cancer patients*

| | Patients with bronchial carcinoma: On diagnosis | | | | |
|---|---|---|---|---|---|
| | All | Later resected | Inoperable | "Clear" after resection | Recurrent or terminal |
| Number of samples | 114 | 22 | 32 | 29 | 29 |
| Correlation[a] of: | | | | | |
| Plasma vitamin C with: | | | | | |
| Buffy-coat vitamin C | 0·45*[b] | 0·36** | 0·61* | 0·36* | 0·33* |
| % Lymphocytes | 0·07 | 0·17 | 0·01 | 0·21 | −0·01 |
| % Monocytes | 0·09 | 0·14 | 0·08 | −0·03 | 0·17 |
| Buffy-coat vitamin C with: | | | | | |
| % Lymphocytes | 0·11[c] | 0·13[d] | 0·13 | 0·12[e] | 0·10 |
| % Monocytes | 0·12[d] | 0·00 | 0·17 | 0·08 | 0·33**[f] |

[a] Kendall's rank-order correlation coefficient, $\tau$.
[b] For all 154 samples $\tau = 0{\cdot}42$, $P < 0{\cdot}001$.
[c] Lymphocytes < 25% $\tau = +0{\cdot}16$**, ⩾25% $\tau = -0{\cdot}08$ n.s.
[d] Reduced by controlling for plasma vit C.
[e] Lymphocytes < 25% $\tau = +0{\cdot}56$**, ⩾25% $\tau = -0{\cdot}38$**.
[f] $\tau = 0{\cdot}29$, $P < 0{\cdot}07$ after controlling for plasma vit C.
*, **, $P < 0{\cdot}01$, $< 0{\cdot}05$.

FIG. 3.—Buffy-coat vitamin C and percentage of lymphocytes in peripheral blood in patients apparently free of disease 6 months to 20 years after resection.

nuclear cell (Table VI) but this was reduced by controlling for plasma vitamin C in the series as a whole and in most of the subgroups.

When this was examined more closely, the correlations of buffy-coat vitamin C with monocytes differed according to the tumour load. Buffy-coat vitamin C was *not* related to the percentage of monocytes in patients who were subsequently resected (Table VI) but tended to be in those with a greater tumour load (inoperable $\tau = 0{\cdot}17$, N.S.; recurrent etc. $\tau = 0{\cdot}33$, $P < 0{\cdot}05$). These patients did *not* have higher proportions of monocytes in peripheral blood, and the correlation was not altered by allowing for plasma vitamin C levels (inoperable $\tau = 0{\cdot}15$; recurrent etc. $\tau = 0{\cdot}29$).

In the case of lymphocytes, a dual relationship between the percentage in peripheral blood and the buffy-coat vitamin C lay behind the apparently poor correlation. In patients who were apparently clear of disease months to years after resection, there was a *direct* relationship between buffy-coat vitamin C and the percentage of lymphocytes in samples with lower lymphocyte counts (Fig. 3), and an *inverse* correlation in those showing relative lymphocytosis ($\geqslant 25\%$; Wintrobe, 1974). Each of these correlations reached significance if an arbitrary division at 25% lymphocytes was imposed ($< 25\%$ lymphocytes, $n = 12$, $\tau = +0{\cdot}56$, $P < 0{\cdot}02$; $\geqslant 25\%$ lymphocytes, $n = 17$, $\tau = -0{\cdot}38$, $P < 0{\cdot}04$) and were unaffected by controlling for plasma values ($< 25\%$, $\tau = +0{\cdot}54$; $\geqslant 25\%$, $\tau = -0{\cdot}40$). The "unreactive" group (lymphocytes $< 25\%$) included most of the patients resected many years before (median 3·5 years after resection) in whom recurrence is relatively unlikely. In contrast, the "reactive" group (lymphocytes $\geqslant 25\%$) had a median follow-up of less than a year and included 5/6 patients tested within 6 months of operation. Reynolds *et al.* (1979) calculated that the recurrence rate for Stage 1 and 2 lung cancer was 2·7% per month for the first 18 months; hence a number of the "reactive" group would be expected to have subclinical disease because of the relatively short follow-up. Higher lymphocyte counts were significantly associated with a shorter follow-up in patients clinically free of disease after resection ($\tau = 0{\cdot}33$, $P < 0{\cdot}02$).

If the lines characteristic of the "reactive" and "unreactive" groups were applied to the plots of buffy-coat vitamin C values and lymphocyte counts from other groups of bronchial-carcinoma patients, the left-hand line (unreactive) described most patients with inoperable or recurrent disease, except that, as expected, more of these patients (inoperable 7/32, recurrent etc. 5/29) had low lymphocyte counts ($< 10\%$) in comparison with the "clear" group (1/32, $\chi^2$ 4·8, $P < 0{\cdot}05$).

In the series as a whole, there was a positive correlation between buffy-coat vitamin C and lymphocyte count only in patients without lymphocytosis (Table VI). Higher lymphocyte counts were associated with lower tumour load ($\tau = 0{\cdot}28$, $P < 0{\cdot}001$) and with resectability ($\tau = 0{\cdot}28$, $P < 0{\cdot}02$). Relative monocytosis was also associated with resectability ($\tau = 0{\cdot}24$, $P < 0{\cdot}05$) but these correlations were only marginally increased by considering mononuclear cells as a whole (tumour load $\tau = 0{\cdot}29$, $P < 0{\cdot}001$; resectability $\tau = 0{\cdot}32$, $P < 0{\cdot}01$). The correlations with absolute lymphocyte counts were weaker.

The anomalies of the relationship between buffy-coat vitamin C and the percentages of mononuclear cells in peri-

FIG. 4.—(a) Buffy-coat vitamin C; and (b) Mononuclear-cell vitamin C, plotted against plasma vitamin C in 14 patients tested at diagnosis. ○ Subsequently resected. ● Inoperable.

FIG. 5.—Mononuclear-cell vitamin C and lymphocytes in peripheral blood in 14 patients tested at diagnosis. ○ Subsequently resected. ● Inoperable.

pheral blood were examined further by estimating mononuclear-cell vitamin C, in addition to the other parameters, in 14 patients at the time of diagnosis. In these patients the buffy-coat values followed the same pattern as in the series as a whole, showing both a similar strength of correlation with plasma vitamin C ($\tau = 0{\cdot}46$, $P < 0{\cdot}03$) and the tendency for the buffy-coat value to be maintained in the presence of lower plasma values (Fig. 4a) previously described (Basu & Schorah, 1982). The mononuclear-cell vitamin C values showed a steeper relationship with plasma vitamin C ($\tau = 0{\cdot}57$, $P < 0{\cdot}004$, Fig. 4b). Patients with lower mononuclear-cell vitamin C values tended to have higher lymphocyte counts (Fig. 5), giving direct support to the reality of the inverse relationship between buffy-coat vitamin C and the lymphocyte count seen in patients with lymphocytosis after resection.

The patients with resectable tumours showed *lower* levels of mononuclear-cell vitamin C than those who were inoperable ($\tau = 0{\cdot}45$, $P < 0{\cdot}05$, Fig. 4b), although the buffy-coat vitamin C values were not significantly different in the two groups (Fig. 4a). In fact, the 7 lowest mononuclear-cell values were all in patients who were subsequently resected, and included those with the 4 highest lymphocyte and 3 highest monocyte percentages in peripheral blood and the 5 lowest plasma values.

The effect of tumour differentiation on vitamin C levels was examined in 101 patients with squamous carcinoma. Poorly differentiated tumours were associated with lower plasma vitamin C levels overall ($\tau = 0{\cdot}19$, $P < 0{\cdot}03$) but this was largely due to stronger correlation within the group of

TABLE VII.—*Assay of total vitamin C in surgical specimens of* 13 *primary lung tumours and in necropsy samples of normal tissue*

| | | N | Vitamin C (mean and s.d.) μg/g tissue | μg/mg protein |
|---|---|---|---|---|
| Primary lung tumours | | 13 | 111·6±55·1 | 1·29±0·62 |
| Normal | Lung | 6 | 58·5±20·4 | 0·87±0·60 |
| | Brain | 4 | 110·7±25·5 | 1·38±0·48 |
| | Muscle | 6 | 14·7±9·3 | 0·22±0·12 |

patients clinically clear of disease after resection ($\tau = 0{\cdot}32$ $P < 0{\cdot}03$).

Assay of vitamin C in 13 surgical specimens of primary lung cancers gave values double that for necropsy samples of normal lung (Table VII). Values for lung, brain and skeletal muscle were similar to those reported in the literature (Basu & Schorah, 1982). The same pattern was evident when results were expressed as a fraction of the weight of the tissue prepared or of the protein content of the homogenate.

Three terminal patients and a 4th patient, in whom the diagnosis of pulmonary tuberculosis was made during the study, were treated with vitamin C (1 g daily x 3, 200 mg daily to 2 weeks); in each case buffy-coat vitamin C rose during treatment. Two patients received vitamin C from the time of resection (1 g daily x 6, 300 mg daily to 3 months); plasma and buffy-coat vitamin C values were in the normal range when retested 6 weeks and/or 6 months after resection (Table VIII).

In this analysis involving the examination of 150 correlations, 2 would have been expected to reach the 1% and 8 the 5% level by chance alone. However, 7 correlations reached the 0·1% level, 12 the 1% and 27 the 5% level of significance (2-tailed). The overall pattern of correlations cannot have arisen by chance alone, though individual correlations may have done so.

### DISCUSSION

Low values for buffy-coat vitamin C have been reported previously in groups of mixed cancer patients and those with advanced disease; a few lung-cancer cases were included. Krasner & Dymock (1974) attributed the low levels primarily to dietary deficiency; Kakar & Wilson (1976) reported that tumour tissue (mainly skin tumours) contained 3 times as much vitamin C as surrounding normal tissue, and postulated that low buffy-coat levels were due to preferential accumulation of vitamin C in the tumour.

Our study confirmed the low levels of plasma and buffy-coat vitamin C in lung-cancer patients in a much larger study, including patients at all stages of the disease, and underlines the complexity of the factors determining the vitamin C content of plasma and leucocytes in cancer

TABLE VIII.—*The effect of supplementation with vit C on plasma and buffy-coat vit C values in* 6 *patients*

| | Plasma vit C | | | | Buffy-coat vit C | | | |
|---|---|---|---|---|---|---|---|---|
| Pt | Pre-treatment | 1 week | 2 weeks | | Pre-treatment | 1 week | 2 weeks | |
| 1 | 0·42 | 0·39 | 0·72 | | 12·9 | 21·6 | 22·0 | |
| 2 | 0·48 | | | | 10·1 | 12·4 | | |
| 3 | 0·15 | 0·24 | | | 8·2 | 20·2 | | |
| 4 | 0·08 | 0·17 | 0·75 | | 11·9 | 20·8 | 38·0 | |
| Pt | Pre-operative | 2 weeks | 6 weeks | 6 months | Pre-operative | 2 weeks | 6 weeks | 6 months |
| 5 | 0·13 | 0·51 | 0·61 | | 16·9 | 27·5 | 29·4 | |
| 6 | 0·21 | | 0·99 | 0·70 | 18·7 | | 32·5 | 45·0 |

Vitamin C regime: 1 g daily x 3 then 200 mg daily for 2 weeks (Patients 1–4), or 1 g daily x 6 then 300 mg daily for 3 months (Patients 5 and 6). Patients 1–3 were suffering from terminal bronchial carcinoma; cases 5 and 6 from bonchial carcinoma, resected after the first sample; in case 4 the diagnosis of pulmonary tuberculosis was made during study.

patients, pointing to additional important factors.

Our findings support those of Krasner & Dymock (1974) in that buffy-coat vitamin C levels in cancer patients were diet-dependent. Seasonal differences were noted throughout the study in agreement with earlier findings from this laboratory (Schorah *et al.*, 1978) and, among interviewed follow-up patients, on a crude assessment, those who ate better diets showed higher plasma and buffy-coat vitamin C levels. In addition, the treatment of terminal-cancer patients, and of patients undergoing resection, with a relatively modest regime of vitamin C supplementation, increased both the plasma and the buffy-coat vitamin C levels in every patient (Table VIII).

However, there are reasons for suspecting that dietary deficiency alone was not responsible for the low vitamin C values found, since levels were lower in post-resection lung-cancer patients who were symptom-free and had been so for months to years, than in healthy controls in the same area. Northern industrial towns are known for poorer vitamin C intakes and plasma concentrations (National Food Survey Committee 1974; Exton-Smith, 1979). With barely adequate vitamin C intake, even a mild increase in utilization would deplete vitamin C reserves. Increased utilization could occur in several ways in these lung-cancer patients.

Contrary to expectation (Pelletier, 1970; Hume & Weyers, 1973), there was no evidence that either infections or current smoking had contributed to the low vitamin C values in lung-cancer patients. Leucocyte counts did not correlate inversely with vitamin C values, and the latter were not lower in patients with recent infections.

The higher vitamin C content of lung tumour than of normal lung supports the proposition of Kakar & Wilson (1976) that increased accumulation of vitamin C in the tumour itself contributes to vitamin C depletion. The apparent link between poorly differentiated tumours (which tend to grow faster) and low plasma vitamin C in our data could also be construed in this way, but may be spurious, since it was strongest in the patients believed to be tumour-free after resection.

Two other factors also seem to have contributed: increased utilization of vitamin C in repair after major surgery and the chronic utilization in defence against residual or recurrent disease. Vitamin C is known to be used in the production of connective tissue required in repair processes (Tuderman *et al.*, 1977; Nambisan & Kurup, 1975). Accentuation of depletion by major surgery would be expected (Crandon *et al.*, 1958; Shukla, 1969; Irwin & Hutchings, 1976), and was demonstrated in the vitamin C values recorded for patients tested in the 6 months after resection, and in the tendency for the operation of pneumonectomy to be associated with lower levels during follow-up, perhaps connected with the substantial "repair" involved in organizing the clot in the pneumonectomy space. What was unexpected was that the levels remained low for many years in the apparent absence of recurrence.

Our data indicated that utilization of vitamin C in tumour resistance also played a part. Allowing for the contribution of platelets to buffy-coat vitamin C, but not substantially to mononuclear-cell vitamin C, a proportionate increase in buffy-coat vitamin C with increase in the proportion of mononuclear cells in peripheral blood would be expected, since normal mononuclear cells contain 2–3 times as much vitamin C as polymorphs (unpublished). This was not seen in all the lung-cancer patients but *was* seen in those without lymphocytosis. In the patients apparently free of disease after resection, a *direct* relationship in patients with low levels of lymphocytes turned into an inverse relationship in those with relative lymphocytosis. The dissociation occurred at the upper end of the normal range for lymphocytes, as cited by Wintrobe (1974), and divided the patients into two groups also differing in the likelihood of undetected recurrent disease because of their very different lengths of follow-up since resection. In neither group was the correlation affected by controlling for plasma vitamin C. This suggests that lower lymphocyte counts and a direct correlation with buffy-coat vitamin C reflected an "unreactive" state, possibly because these patients had no tumour to "react" to, and that their plasma and buffy-coat vitamin C values still showed evidence of incomplete recovery after the depletion of the tumour-bearing and postoperative repair phases.

There were echoes of this "unreactive" pattern in patients with substantial tumour load, but in this case there were more patients with extreme low values for both lymphocyte counts and buffy-coat vitamin C. Lymphocyte counts below the normal range were always accompanied by low buffy-coat and plasma vitamin C levels, raising the possibility that nutrient deficiency may contribute to the decrease in circulating lymphocytes reported in patients entering the terminal phase (Chretien *et al.*, 1973; Anthony *et al.*, 1975).

In contrast, patients with higher lymphocyte counts showed an *inverse* relationship of lymphocyte count with the buffy-coat vitamin C ("reactive" pattern), not affected by controlling for plasma values; lymphocytes contributing less to the buffy-coat vitamin C with increasing lymphocytosis. This is unlikely to have been caused by inadequate intake alone and is difficult to explain, unless vitamin C is being used by some mechanism associated with the lymphocytosis. Lymphocytosis was first reported to be associated with resistance to spontaneous tumours by Murphy (1926) and has been linked to longer survival of inoperable patients with squamous bronchial carcinoma, the correlation being unaffected by controlling for other factors (Anthony *et al.*, 1981). Significantly less vitamin C was present in the mononuclear-cell fraction of presenting patients who were subsequently resected than in those of patients of poorer prognosis, but there was less difference in buffy-coat vitamin C. The better-prognosis group also had higher lymphocyte counts. This would be explained if utilization of vitamin C by lymphocyte-related mechanisms in patients with some measure of resistance to their tumours led to more general cell and plasma depletion. Low buffy-coat vitamin C has been reported in conditions such as rheumatoid arthritis (Mullen & Wilson, 1976) and asthma (Olusi *et al.*, 1979), in which chronic immune reactions are involved. Evidence for the utilization of vitamin C in the defence against cancer has not previously been presented.

Although the obvious conclusion to the finding that vitamin C is utilized in lymphocyte-related mechanisms in patients with lung cancer would be to assume that vitamin C supplementation to maintain normal buffy-coat vitamin C levels would increase resistance to tumour extension, this cannot be assumed, and our data offers no evidence as to whether this would be so. Regression of tumours in guinea-pigs given only just enough vitamin C to prevent death from scurvy, and enhanced growth with very high vitamin C dosage (Migliozzi, 1977), indicate that tumours, like other tissues, require vitamin C for growth. We have no evidence that vitamin C depletion depressed the growth rate of lung cancer in our patients; if this were so, supplementation could also benefit the tumour. On the other hand, vitamin C depletion has been reported to interfere both with effector capacity and with the orchestration of the immune response. In "reactive" patients with low vitamin C, one would anticipate that supplementation would allow fuller expression of cell-mediated immunity and phagocytosis, restoring some degree of tumour control where this had been present; but in "unreactive" tumour-bearing patients, this would only be so if vitamin C depletion were responsible for the state of "unreactivity".

Our data are unable to predict the overall effect of supplementation with vitamin C, which could only satisfactorily be tested in the setting of a clinical trial. Our results suggest, however, that relatively modest dosage with vitamin C would be adequate to prevent vitamin C depletion, and that this level of dosage would be worth testing.

We should like to thank our clinical colleagues, particularly Mr D. Walker and Dr N. Cooke for their collaboration, Dr T. Muller for his help, Miss M. Liddell for preparing the tissue, N. Habibzadeh and Mrs B. Peel for technical assistance, and Hoffmann–La Roche and Co., Basle, and the Yorkshire Cancer Research Campaign for financial support.

### REFERENCES

ANDERSON, R. (1981) Assessment of oral ascorbate in three children with chronic granulomatous disease and defective neutrophil motility over a two-year period. *Clin. Exp. Immunol.*, **43**, 180.

ANDERSON, R. & DITTRICH, O. C. (1979) Effects of ascorbate on leucocytes. *S. Afr. Med. J.*, **56**, 476.

ANTHONY, H. M., KIRK, J. A., MADSEN, K. E., MASON, M. K. & TEMPLEMAN, G. H. (1975) E and EAC rosetting lymphocytes in patients with carcinoma of bronchus. II. A sequential study of thirty patients: Effect of BCG. *Clin. Exp. Immunol.*, **20**, 41.

ANTHONY, H. M., MADSEN, K. E., MASON, M. K. & TEMPLEMAN, G. H. (1981) Lung cancer, immue status, histopathology and smoking. Is oat cell carcinoma lymphodependent? *Br. J. Dis. Chest*, **75**, 40.

BASU, T. S. & SCHORAH, C. J. (1982) *Vitamin C in Health and Disease*. London: Croom Helm. pp. 84, 66–82, 32, 15.

BØYUM, A. (1968) Separation of leucocytes from blood and bone marrow. *Scdnd. J. Clin. Lab. Invest.*, **21** (Suppl), 97.

CAMERON, E., PAULING, L. & LEBOVITZ, B. (1979) Ascorbic acid and cancer: A review. *Cancer Res.*, **39**, 663.

CHRETIEN, J. H. & GARAGUSI, V. F. (1973) Correction of cortico-steroid induced defects of PMN function by ascorbic acid. *J. Reticuloendoth. Soc.*, **14**, 280.

CHRETIEN, P. B., CROWDER, W. L., GERTNER, H. R., SAMPLE, W. F. & CATALONA, W. J. (1973) Correlation of preoperative lymphocyte reactivity with the clinical course of cancer patients. *Surg. Gynaecol. Obst.*, **136**, 380.

CRANDON, J. H., LANDAU, B., MIKAL, S., BALMANNO, J., JEFFERSON, M. & MAHONEY, N. (1958) Ascorbic acid economy in surgical patients as indicated by blood ascorbic acid levels. *N. Engl. J. Med.*, **258**, 105.

DALLEGRI, F., LANZI, G. & PATRONE, F. (1980) Effects of ascorbic acid on neutrophil locomotion. *Int. Archs. Allergy Appl. Immunol.*, **61**, 40.

DENSON, K. W. & BOWERS, E. F. (1961) The determination of ascorbic acid in white blood cells. *Clin. Sci.*, **21**, 157.

DI PAOLA, M., BERTOLOTTI, A., COLIZZA, S. & COLI, M. (1977) Histology of bronchial carcinoma and regional lymph nodes as putative immune response of the host to the tumour. *J. Thor. Cardiovasc. Surg.*, **73**, 531.

EXTON-SMITH, A. N. (1979). *Nutrition and Health in Old Age.* H.M.S.O. DHSS.

GOTH, A. & LITTMANN, I. (1948) Ascorbic acid content of human cancer tissue. *Cancer Res.*, **8**, 349.

HUME, R. & WEYERS, E. (1973) Changes in leucocyte ascorbic acid during the common cold. *Scot. Med. J.*, **18**, 3.

HUME, R., WEYERS, E., ROWAN, T., REID, D. S. & HILLIS, W. S. (1972) Leucocyte ascorbic acid levels after acute myocardial infarction. *Br. Heart J.*, **34**, 238.

IOACHIM, H. L., DORSETT, B. H. & PALUCH, E. (1976) The immune response at the tumour site in lung carcinoma. *Cancer*, **38**, 2296.

IRWIN, M. I. & HUTCHINGS, B. K. (1976) A conspectus of research on vitamin C requirements of man. *J. Nutr.*, **106**, 821.

KAKAR, S. C. & WILSON, C. W. M. (1976) Ascorbic acid values in malignant disease. *Proc. Nutr. Soc.*, **35**, 9A.

KALDEN, J. R. & GUTHY, E. A. (1972) Prolonged skin allograft survival in vitamin C-deficient guinea pigs. *Eur. Surg. Res.*, **4**, 114.

KAUFMANN, M., WIRTH, K., SCHEURER, J., ZIMMERMAN, A., LUSCIETI, P. & STJERNSWARD, J. (1977) Immunomorphological lymph node changes in patients with operable bronchogenic squamous cell carcinoma. *Cancer*, **39**, 2371.

KOLB, E. & MULLER, E. (1979) Local responses in primary and secondary human lung cancers. II. Clinical considerations. *Br. J. Cancer*, **40**, 410.

KRASNER, N. & DYMOCK, I. W. (1974) Ascorbic acid deficiency in malignant disease: A clinical and biochemical study. *Br. J. Cancer*, **30**, 142.

MANZELLA, J. P. & ROBERTS, N. J. (1979) Human macrophage and lymphocyte responses to mitogen stimulation after exposure to influenza virus ascorbic acid and hyperthermia. *J. Immunol.*, **123**, 1940.

MIGLIOZZI, J. A. (1977) Effect of ascorbic acid on tumour growth. *Br. J. Cancer*, **35**, 448.

MULLEN, A. & WILSON, C. W. M. (1976) The metabolism of ascorbic acid in rheumatoid arthritis. *Proc. Nutr. Soc.*, **35**, 8A.

MURPHY, J. B. (1926) The lymphocyte in resistance to tissue grafting, malignant disease and tuberculous infection: An experimental study. *Rockefeller Inst. Med. Res. Monogr.*, 21.

NAMBISAN, B. & KURUP, P. A. (1975) Ascorbic acid and glycosaminoglycans and lipid metabolism in guinea pigs fed normal and atherogenic diets. *Atherosclerosis*, **22**, 447.

NATIONAL FOOD SURVEY COMMITTEE (1974) *Household Food Consumption and Expenditure, 1972.* London: H.M.S.O.

NIE, N. H., HULL, C. H., JENKINS, J. G., STEINBRUNNER, K. & BENT, D. H. (1975) *Statistical Package for the Social Sciences*, (2nd ed). New York: McGraw Hill, pp. 288, 302.

OLUSI, S. O., OJUTIKU, O. O., JESSOP, W. J. E. & IBOKO, M. I. (1979) Plasma and white blood cell ascorbic acid concentrations in patients with bronchial asthma. *Clin. Chim. Acta.*, **92**, 161.

PELLETIER, O. (1970) Vitamin C status of cigarette smokers and non-smokers. *Am. J. Clin. Nutr.*, **23**, 520.

REBORA, A., DALLEGRI, F. & PATRONE, F. (1980) Neutrophil dysfunction and repeated infections: Influence of levamisole and ascorbic acid. *Br. J. Dermatol.*, **102**, 49.

Reynolds, R. D., Pajak, T. F., Bateman, J. R. & 6 others (1979) Considerations in designing and analysing surgical adjuvant study in resected Stage I and II carcinoma of the lung. *Cancer*, **44**, 1201.

Schorah, C. J., Zemroch, P. J., Sheppard, S. & Smithells, R. W. (1978) Leucocyte ascorbic acid and pregnancy. *Br. J. Nutr.*, **39**, 139.

Shukla, S. P. (1969) Plasma and urinary ascorbic acid levels in the post-operative period. *Experientia*, **25**, 704.

Thomas, W. R. & Holt, P. G. (1978) Vitamin C and immunity: An assessment of the evidence. *Clin. Exp. Immunol.*, **32**, 370.

Tuderman, L., Myllyla, R. & Kivirikko, K. I. (1977) Mechanism of the prolyl hydroxylase reaction. *Eur. J. Biochem.*, **80**, 341.

Wintrobe, M. M. (1974) *Clinical Haematology*, (7th ed.) Philadelphia: Lea & Febiger, p. 1801.

Zweiman, B., Besdine, R. W. & Hildreth, E. A. (1966) The effect of the scorbutic state on tuberculin hypersensitivity in the guinea pig. II. *In vitro* mitotic response of lymphocytes. *J. Immunol.*, **96**, 672.